# Insights into the origin of the nuclear localization signals in conserved ribosomal proteins

**DOI:** 10.1038/ncomms8382

**Published:** 2015-06-11

**Authors:** Sergey Melnikov, Adam Ben-Shem, Gulnara Yusupova, Marat Yusupov

**Affiliations:** 1 Strasbourg University, 4 Rue Blaise Pascal, 67081 Strasbourg, France; 2 Institute of Genetics and Molecular and Cellular Biology, 1 Rue Laurent Fries, 67400 Illkirch-Graffenstaden, France; 3 Department of Molecular Biophysics and Biochemistry, Yale University, 266 Whitney Avenue, New Haven, Connecticut 06511, USA; 4 CNRS, 1 Rue Laurent Fries, 67400 Illkirch-Graffenstaden, France

## Abstract

Eukaryotic ribosomal proteins, unlike their bacterial homologues, possess nuclear localization signals (NLSs) to enter the cell nucleus during ribosome assembly. Here we provide a comprehensive comparison of bacterial and eukaryotic ribosomes to show that NLSs appear in conserved ribosomal proteins via remodelling of their RNA-binding domains. This finding enabled us to identify previously unknown NLSs in ribosomal proteins from humans, and suggests that, apart from promoting protein transport, NLSs may facilitate folding of ribosomal RNA.

## Abstract (web summary)

Eukaryotic ribosomal proteins contain nuclear localization signals (NLSs) that their bacterial counterparts lack. Here the authors compare homologous proteins from bacterial and eukaryotic ribosomes to show how NLSs could emerge in the course of evolution, and use this knowledge to identify novel NLSs.

## Graphical abstract

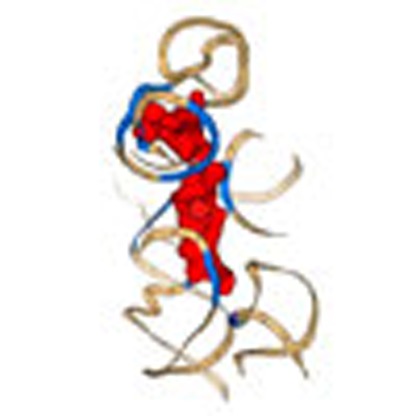

Ribosomes share similar architecture in all branches of life, but their assembly pathways are dramatically divergent across species, particularly between bacteria and eukaryotes. In eukaryotes, ribosome assembly requires an intricate trafficking of ribosomal proteins; produced in the cytoplasm, they first enter the cell nucleus and accumulate in the nucleolus before they associate into nascent ribosomes^1^. Therefore, eukaryotic ribosomal proteins are thought to harbour nuclear/nucleolar localization signals (NLSs) – short, predominantly basic stretches of amino acids that trigger active transport of proteins to the nucleus^2^,^3^,^4^. Given the ancient origin of ribosomes, the question arises – how did NLSs emerge in conserved ribosomal proteins? Were similar motifs present in prokaryotic proteins, and if not, what structural changes were required to evolve the NLSs? Subsequently, how might these changes influence the overall ribosome structure?

To address these questions, we provide here a comprehensive comparison of homologous proteins from bacterial and eukaryotic ribosomes and revise available data about the NLSs in ribosomal proteins. Firstly, we show that NLSs emerged in conserved ribosomal proteins via remodelling of their RNA-binding domains. Surprisingly, this remodelling occurred mainly in the highly conserved interior of the ribosome. In the interior, NLSs form extensive and selective interactions with ribosomal RNA (rRNA), binding predominantly to single-stranded helical junctions, which point to a possible role of the NLSs in rRNA folding. Finally, we use these structural observations to identify novel NLSs in human ribosomal proteins uS12 and uL24.

## Results

### NLSs altered RNA-binding domains of the conserved proteins

**Figure 1:**
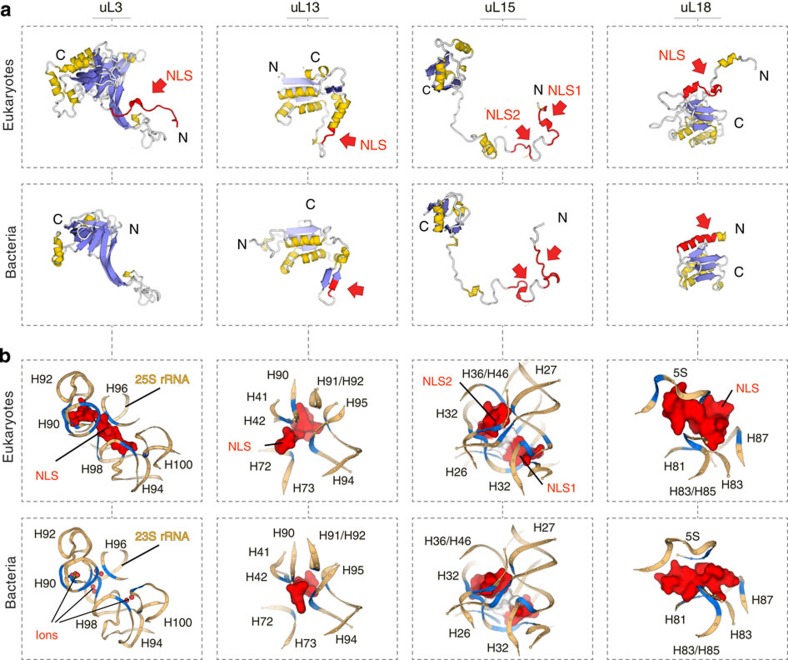
Mapping nuclear/nucleolar localization signals (NLSs) within the ribosome structure reveals their common structural features and provides an insight into their evolutionary origin. (**a**) Crystal structures of four pairs of homologous proteins from 70S *E. coli* and 80S *S. cerevisiae* ribosomes: proteins are coloured according to the secondary structure, with red colour and red arrows pointing to NLSs of eukaryotic proteins (top panels) and to corresponding positions in bacterial homologues (bottom panels). NLSs reside within non-globular extensions of eukaryotic proteins with substantially remodelled secondary and tertiary structure compared with analogous protein segments in bacterial ribosomal proteins. (**b**) Fragments of the ribosome interior with a zoom on interactions between NLSs and rRNA within the eukaryotic ribosome (top panels) and corresponding segments of bacterial ribosome structure (bottom panels); nucleotides, which contact ribosomal proteins and ions/water molecules (shown as spheres), are in blue; labels correspond to 23S/25S rRNA helices. When ribosomal proteins are incorporated into the ribosome, NLSs are buried in the rRNA: compared with bacterial ribosomes, NLSs structurally replace non-globular extensions of bacterial proteins or magnesium ions/water in the ribosome interior and form similar stabilizing contacts with single-stranded helical junctions of conserved rRNA, suggesting a role of NLSs in rRNA folding.

To gain an insight into the evolutionary origin of the NLSs in ribosomal proteins, we first mapped previously identified NLSs in the crystal structure of the eukaryotic ribosome from budding yeast *Saccharomyces cerevisiae* (*S. cerevisiae*), analysed their structure, and compared them to the corresponding segments in homologous proteins from the *Escherichia coli (E. coli)* ribosome (Methods). In total, we analysed twelve NLSs from ten conserved ribosomal proteins (Supplementary Table 1)^2^,^5^,^6^,^7^,^8^,^9^,^10^,^11^,^12^,^13^. We found both that all the NLSs of ribosomal proteins reside within non-globular extensions of rRNA-binding domains and that these NLS-carrying extensions have different structures in eukaryotes and in bacteria. For instance, NLSs of eukaryotic proteins uS3, uS4, uL13, uL15 and uL18 reside within the extensions that overlap with those of bacterial proteins, but adopt different secondary and tertiary structures (Fig. 1a, Supplementary Fig. 1). This finding was surprising, both because these extensions have similar size and charge in bacteria and eukaryotes and were previously assigned as conserved, according to sequence alignments^14^,^15^,^16^. Other NLSs reside within rRNA-binding extensions that are absent in bacterial proteins – as sequence alignments had shown for proteins uS8, uL3 (ref. 2), uL18 (ref. 6), uL23 (ref. 13) and uL29 (ref. 7; (Fig. 1a, Supplementary Fig. 1). Taken together, this comparison illustrated that, despite high content of basic residues in ribosomal proteins, particularly at their rRNA-binding interface, the NLSs or similar motifs are absent in bacteria and apparently emerged via remodelling of the rRNA-binding domains of conserved ribosomal proteins.

### NLSs maintain conserved folds of the rRNA

To understand how the NLSs were accommodated in the conserved core of the ribosome, we analysed their surroundings and interactions within the ribosome interior. Compared with bacterial ribosomes, the NLSs structurally replace extensions of homologous proteins (uS3, uS8, uL13, uL15 and uL18) or magnesium ions and water (uL3, uL23, uL29 and uS2) and form extensive contacts with rRNA (Fig. 1b, Supplementary Fig. 1). In total, they establish ∼260 salt bridges, hydrogen bonds and stacking interactions. Remarkably, interactions between the rRNA and NLSs have two common tendencies. Firstly, although ribosomal proteins form most of their contacts with rRNA helices, which are thought to play a role in the recruitment of ribosomal proteins to the nascent rRNA transcript^17^, the NLSs bind predominantly to single-stranded rRNA. Thus, ∼96% of their contacts are established with helical junctions, internal loops, or helix–loop borders (Fig. 1b, Supplementary Table 1). Since the structure of single-stranded rRNA segments governs three-dimensional packing of RNA helices, these contacts suggest that NLSs may participate in the rRNA folding. Secondly and most unexpectedly, we found that the NLSs bind predominantly to highly conserved rRNA, whose structure is nearly invariant in both eukaryotes and bacteria, as exemplified by proteins uL3, uL13, uL15 and uL18 (Fig. 1b, Supplementary Fig. 1). This observation challenges the current view that conserved components of the ribosome are mainly divergent at the cytoplasm-exposed surface^14^,^15^,^16^, and illustrates that substantial changes in conserved ribosomal proteins are also present between the universally conserved ribosomal cores of eukaryotes and bacteria.

### Structure-guided search for novel NLSs in ribosomal proteins

**Figure 2:**
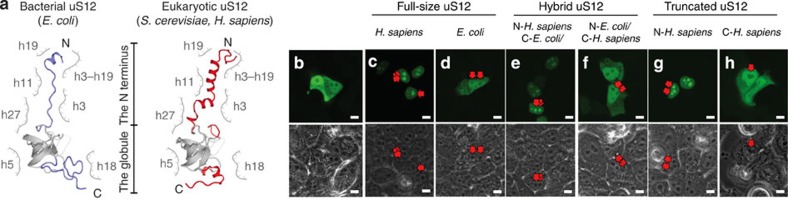
Different structure of homologous ribosomal proteins within highly conserved rRNA pockets may point to the location of unknown nuclear localization signals. Exemplified by identification of NLS in human ribosomal protein uS12. (**a**) Structure of protein uS12, coloured according to structural conservation: conserved fold is shown in grey and bacteria- and eukaryote-specific in blue and red, respectively; surrounding rRNA is shown schematically with labels indicating 16S/18S rRNA helices. Protein uS12 is one of the 15 ribosomal proteins in which extensions have different folds in bacteria and eukaryotes, despite being bound to nearly identical rRNA cavities of bacterial and eukaryotic ribosomes. (**b**–**h**) eGFP fluorescence (top panels) and phase-contrast (bottom panels) snapshots of human cell line HEK293, which express eGFP fusions of human or *E. coli* protein uS12. Arrows point to nucleoli. All scale bars represent 10 μm. For each sample, eGFP localization was examined in 200 cells. Cells, in which eGFP distribution pattern was common for >95% of the analysed population, were used for imaging. The experiments were replicated three times. (**b**) eGFP alone (a negative control). (**c**) Human uS12–eGFP fusion. (**d**) *E. coli* uS12–eGFP fusion. (**e**) A protein hybrid carrying the N terminus of human uS12 and the globular domain of *E. coli* uS12. (**f**) A protein hybrid carrying the N terminus of *E. coli* uS12 and the globular domain of human uS12. (**b**–**h**) Identifying NLSs within human uS12 shows that its N-terminal extension (N-*H. sapiens*) carries NLS activity – by contrast to analogous extension in bacterial uS12 (N-*E. coli*).

Having elucidated previously unknown consensus structural features of NLSs of ribosomal proteins, we next endeavoured to use our structural observations to uncover unknown NLSs. For this purpose, we examined how many homologous proteins have different folds of their rRNA-binding domains within similar rRNA cavities of bacterial and eukaryotic ribosomes. We preformed structural comparison of all homologous proteins from eukaryotic (*S. cerevisiae*) and bacterial (*E. coli*) ribosomes and, where necessary, analysed surrounding of ribosomal proteins in the ribosome structure (Supplementary Online Methods). This approach allowed us to resolve ambiguity between previous sequence^15^,^16^ and secondary structure alignments^14^ and correct numerous mistakes in the correspondence between the residues of uS3, uS4, uS11, uS12, uS13, uS15, uS19, uL2, uL3, uL4, uL5, uL9, uL13, uL15 and uL18 (Supplementary Fig. 2). In total, we found that 27 out of 32 conserved proteins possess differently folded segments within their rRNA-binding domains between bacteria and eukaryotes, despite overall conserved structure of the adjacent rRNA: in fifteen proteins, seemingly conserved extensions have different fold in bacteria and eukaryotes, and, in seventeen proteins, extensions differ in size despite the high conservation of the surrounding rRNA (Supplementary Fig. 2). To verify if these structural differences could indeed point to the location of the NLSs, we selected proteins uS12 and uL24, whose rRNA-binding domains are among the most divergent in the small and the large ribosomal subunits, respectively (Fig. 2a, Supplementary Fig. 3). Next, we expressed uS12 fused to enhanced green fluorescent protein (eGFP) in human cells. Unlike a control eGFP sample (Fig. 2b), human uS12–eGFP fusion accumulated in the nucleoli, indicating the presence of NLS (Fig. 2c, Supplementary Fig. 4a). By contrast, the *E. coli* uS12–eGFP fusion was distributed between the nucleus and the cytoplasm and largely excluded from the nucleoli, consistent with the absence of NLSs in bacteria (Fig. 2d, Supplementary Fig. 4b). Next, we replaced the N terminus of *E. coli* uS12 with the N terminus of human uS12, a structure that is highly divergent between the two organisms despite high conservation of rRNA (Fig. 2a). We found that the resulting chimeric protein gained nucleolar accumulation in human cell lines HEK293 (Fig. 2e, Supplementary Fig. 4c). Subsequently, when the extension of human uS12 was replaced by the N terminus of its bacterial homologue, the resulting protein was no longer able to accumulate in the nucleolus (Fig. 2f, Supplementary Fig. 4d). Finally, upon examining isolated individual domains we showed that the N terminus of human uS12 alone is sufficient for the nucleolar accumulation of eGFP (Fig. 2g), whereas the globular domain alone is insufficient (Fig. 2h), indicating that the N-terminal segment indeed carries the NLS activity. Similarly, we identified the NLSs of ribosomal protein uL24, residing within structurally diverged N- and C-termini of this protein (Supplementary Fig. 3).

## Discussion

Collectively, our analysis revealed that the NLSs of conserved ribosomal proteins reside within highly diverged rRNA-binding domains and have extensive contacts with the rRNA. These contacts suggest that having evolved NLSs at the interface with conserved rRNA allows to use the NLSs to not only promote protein trafficking, but also to facilitate rRNA folding during ribosome biogenesis, thereby coordinating delivery of ribosomal proteins to the nascent rRNA with the rRNA folding. This notion is indirectly supported by recent studies of the ribosome biogenesis in bacteria and yeast^18^,^19^,^20^. For instance in *E. coli*, deletion of the N-terminal extension of protein uS12 causes defects of the 16S rRNA architecture and provokes lethality^18^. Since eukaryotic uS12 forms similar contacts with the 18S rRNA, it is possible that its N-terminal and NLS-carrying extension could also promote a proper folding of the 18S rRNA. In another case study in yeast, deletion of protein uL24 was recently shown to cause abnormal folds of the 25S rRNA^19^. Since the contacts between uL24 and rRNA are mediated largely by the NLS-carrying N- and C-terminal extensions of uL24, it is possible that the NLSs of this protein may carry a dual function by promoting a proper folding of rRNA.

Beyond insights into the ribosome biogenesis, our observations suggest how the nuclear localization signals could have emerged in the course of evolution. Of note, there are only 61 universally abundant proteins, which are present in all forms of life, among which 32 are conserved ribosomal proteins^21^. Therefore, the future extension of our analysis to a broad range of organisms, including distant eukaryotic and archaeal species, and systematic screening of their NLSs and NLS-binding partners may bring us closer to understanding how cells developed the nucleocytoplasmic transport system and may potentially provide new tools to control the essential process of ribosome biogenesis in all forms of life.

## Methods

### Comparison of bacterial and eukaryotic ribosomal proteins

Structures of 32 pairs of homologous proteins were derived from *E. coli* 70S ribosome (pdbs 4wao-p) and *S. cerevisiae* 80S ribosome (pdbs 3u5b-d) structures, and were initially aligned by the automated flexible FatCat fitting^22^. The alignments were corrected by comparison of a conformation of a polypeptide chain relative to its surrounding within the ribosome interior. By contrast to previous attempts to comprehensively compare bacterial and eukaryotic proteins – by us^14^ and others^15^,^16^ – we identified numerous extensions with unique structure in bacteria and eukaryotes that were mistakenly annotated as conserved due to their similar size in different species and lack of secondary structure and conserved sequence. These extensions were found in proteins uS3, uS4, uS11, uS12, uS13, uS15, uS19, uL2, uL3, uL4, uL5, uL9, uL13, ul15 and uL18 (Supplementary Fig. 2). NLSs of ribosomal proteins were mapped on the *S. cerevisiae* ribosome according to the original studies summarized in (Supplementary Table 1).

### Cloning and constructing of protein mutants and chimeras

Genes, coding for *Homo sapiens* and *E. coli*, uS12 and uL24, were amplified from HeLa S3 (American Type Culture Collection (ATCC)) cDNA library or from a colony of JM109 *E. coli* strain and were later used for cloning or constructing chimeric genes. The primers used in this study were: to clone *H. sapiens* uS12 (residues 1–143) – GCGCCTCGAGATGGGCAAGTGTCGTGGA and GCGCGGATCCCCTTATGATCTTGGTCTTTCCTTC ; the N terminus of *H. sapiens* uS12 (residues 1–41) – GCGCCTCGAGATGGGCAAGTGTCGTGGA and GCGCGGATCCCCAGGGTTGGCCTTTAGGGCTG ; the globular domain of *H. sapiens* uS12 (residues 42–143) – GCGCCTCGAGATGTTTGGAGGTGCTTCTCATGC and GCGCGGATCCCCTTATGATCTTGGTCTTTCCTTC ; *E. coli* uS12 (residues 1–124) – GCGCCTCGAGATGGCAACAGTTAACCAGCT and GCGCGGATCCCCAGCCTTAGGACGCTTCACGC ; the hybrid protein comprising the N terminus of *H. sapiens* uS12 (residues 1–41) and the globular domain of *E. coli* uS12 (residues 24–124) – GCGCCTCGAGATGGGCAAGTGTCGTGGA , ATGCTTCCAGAGGGTTGGCCTTTAGGGCTG , GGCCAACCCTCTGGAAGCATGCCCGCAAAA and GCGCGGATCCCCAGCCTTAGGACGCTTCACGC ; the hybrid protein comprising the N terminus of *E. coli* uS12 (1–23) and the globular domain of *H. sapiens* uS12 (residues 42–143) – GCGCCTCGAGATGGCAACAGTTAACCAGCT , CACCTCCAAACGCAGGCACGTTGCTTTTCG , CGTGCCTGCGTTTGGAGGTGCTTCTCATGCA and GCGCGGATCCCCTTATGATCTTGGTCTTTCCTTC ; *H. sapiens* uL24 (residues 1–127) – GCGCCTCGAGATGAAGTTTAATCCCTTTGT and GCGCGGATCCCCTTCCTGCATCTTCTCAATGG ; the N terminus of *H. sapiens* (residues 1–49) – GCGCCTCGAGATGAAGTTTAATCCCTTTGT and GCGCGGATCCCCGATGGGCATGGATCGCACGT ; the globular domain of *H. sapiens* uL24 globular domain (residues 50–107) – GCGCCTCGAGCGAAAGGATGATGAAGTTCA and GCGCGGATCCCCGCGAGATTTGGCTTTCCGT ; and the C terminus of *H. sapiens* uL24 (residues 108–127) – GCGCCTCGAGCAAGTAGGAAAGGAAAAGGG and GCGCGGATCCCCTTCCTGCATCTTCTCAATGG . All the PCR products were cloned into pEGFP-N1 vector (Promega) between KpnI and XhoI sites. Their sequence was verified via sequencing analysis (GATC Biotech).

### Cell lines, transfection and microscopy

Nucleolar accumulation of ribosomal proteins was examined in human cell line HEK293T (ATCC). In brief, cells were plated on 35 mm glass-bottom dishes (MatTek) at 25–40% confluence and in 24 h were transfected with FuGene 6 (Promega), according to the manufacturers' protocol. Confocal fluorescent images were obtained by a Zeiss LSM510NLO scanning microscope: 8–12 h after transfection cells were rapidly transferred on a microscope stage, and the eGFP-localization pattern was examined in 200 cells. Cells, which eGFP-localization pattern was common for at least 95% of the analysed population, were used for imaging. For each genetic construct, experiments were repeated at least two times.

## Additional information

**How to cite this article:** Melnikov, S. *et al*. Insights into the origin of the nuclear localization signals in conserved ribosomal proteins. *Nat. Commun.* 6:7382 doi: 10.1038/ncomms8382 (2015).

## Supplementary Material

Supplementary Information Supplementary Figures 1-4, Supplementary Table 1 and Supplementary References.
