# Supplementary material for: Insights into the origin of the nuclear localization signals in conserved ribosomal proteins
Source: Nat Commun. 2015 Jun 11;6:7382. doi: 10.1038/ncomms8382 (PMC4490412; doi:10.1038/ncomms8382)
Supplement: Supplementary Information — Supplementary Figures 1-4, Supplementary Table 1 and Supplementary References. [file ncomms8382-s1.pdf]

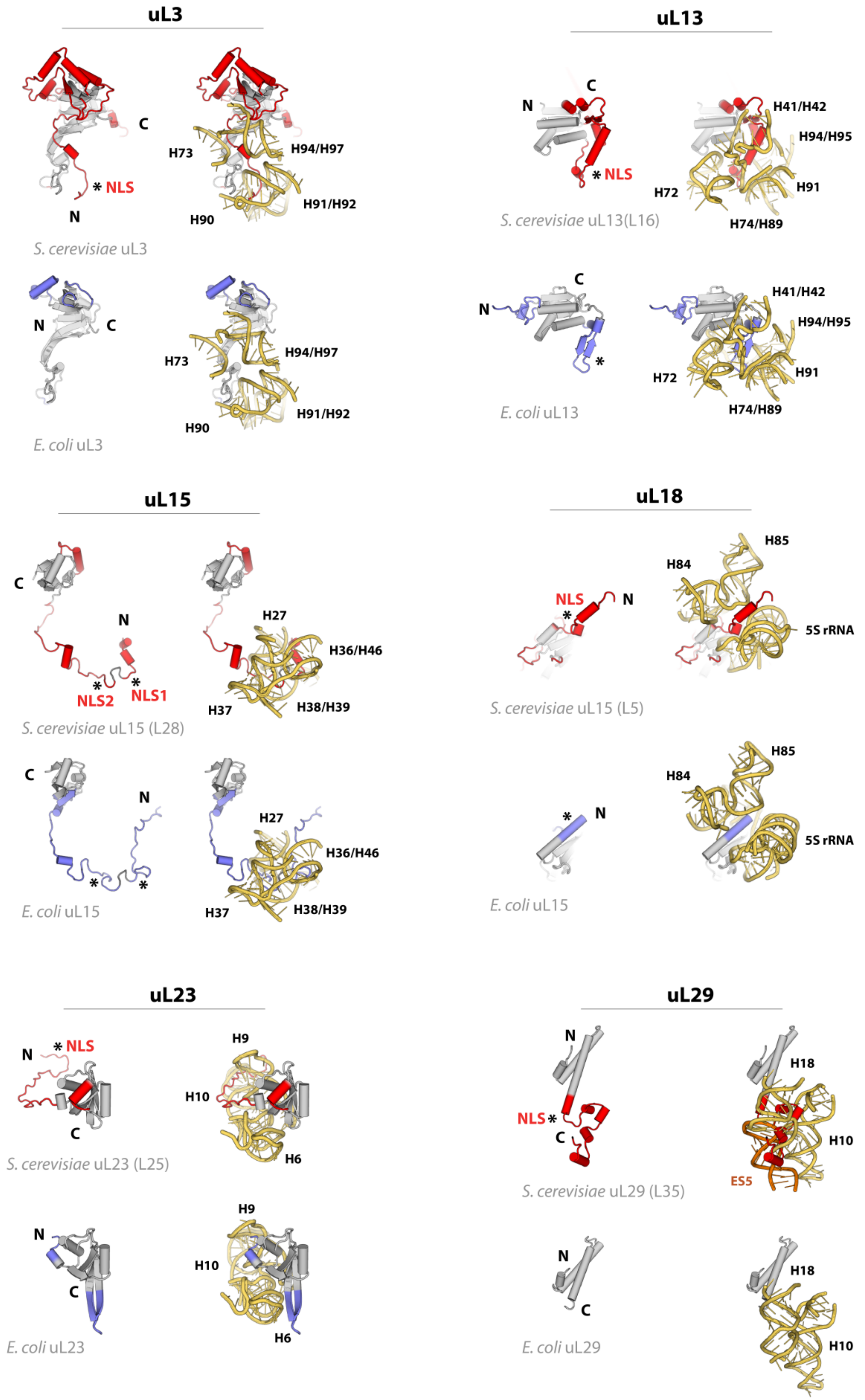
uL3
C
H73
H94/H97
* NLS
H91/H92
N
H90
S. cerevisiae uL3
N
C
H73
H94/H97
H91/H92
H90
E. coli uL3
uL13
C
N
H41/H42
H94/H95
* NLS
H91
H72
S. cerevisiae uL13(L16)
H74/H89
C
H41/H42
N
H94/H95
*
H91
H72
H74/H89
E. coli uL13
uL15
C
N
H27
H36/H46
NLS2
NLS1
H37
H38/H39
S. cerevisiae uL15 (L28)
C
N
H27
H36/H46
H37
H38/H39
E. coli uL15
uL18
H85
H84
NLS
N
5S rRNA
S. cerevisiae uL15 (L5)
H85
H84
N
5S rRNA
E. coli uL15
uL23
N
* NLS
H9
H10
C
H6
S. cerevisiae uL23 (L25)
H9
N
H10
C
H6
E. coli uL23
uL29
N
H18
NLS*
C
H10
ES5
S. cerevisiae uL29 (L35)
N
H18
C
H10
E. coli uL29

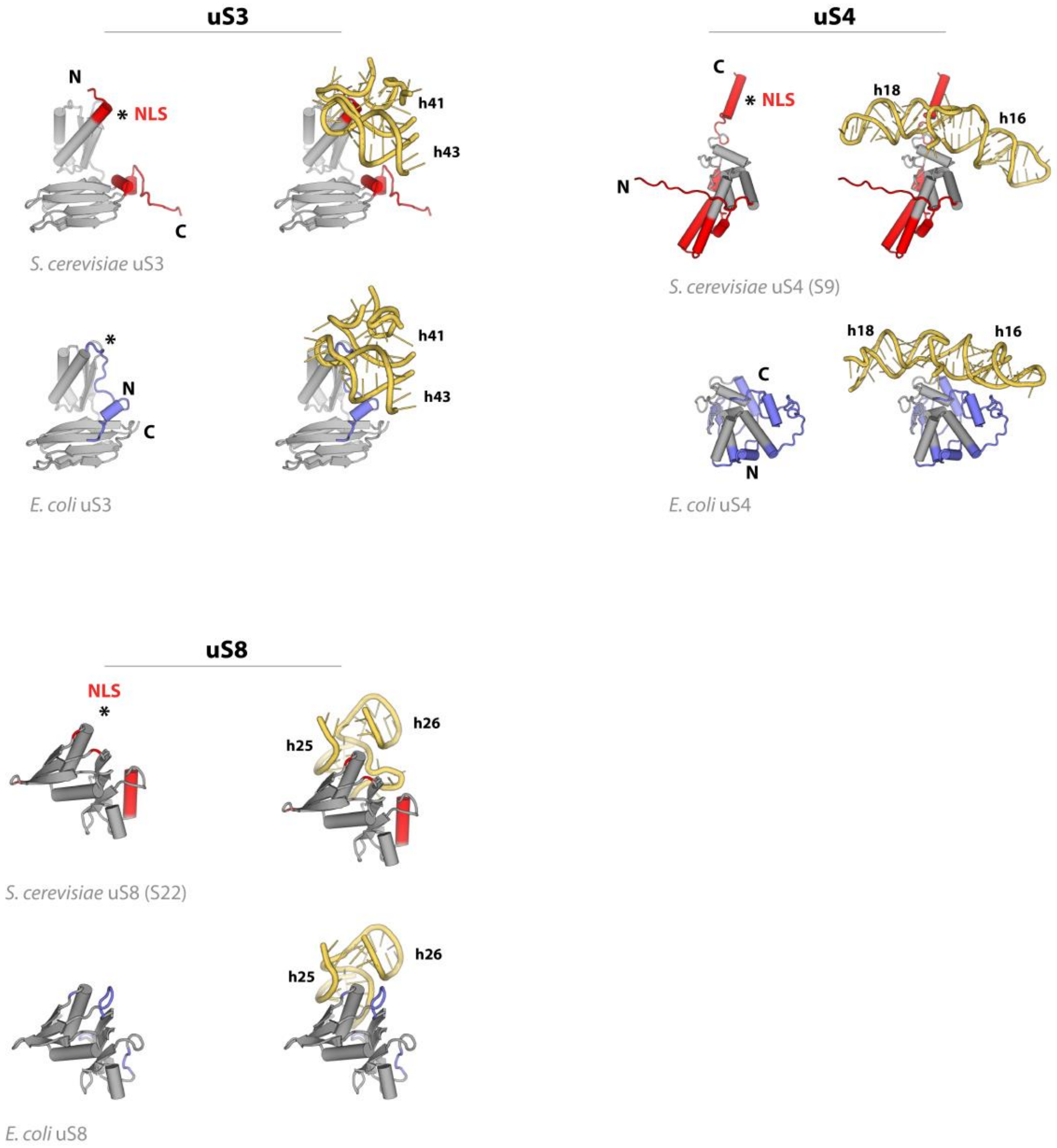

Supplementary Figure 1: Comparison of homologous proteins from eukaryotic and bacterial ribosomes provides an insight into the evolutionary origin of the nuclear localization signals. Panels show pairs of homologous proteins from *E. coli* and *S. cerevisiae* ribosomes and their rRNA-surrounding within the 70S and the 80S ribosome, respectively. Proteins are colored according to their structural conservation, with conserved segments shown in grey, and bacteria- and eukaryote-specific in blue and red, respectively. rRNA is shown as yellow ladders; labels indicate conserved helices of rRNA, or eukaryote-specific expansion segment ES5. Asterisks point to the position of the NLSs in eukaryotic ribosomal proteins and to the corresponding segments in their bacterial homologs.

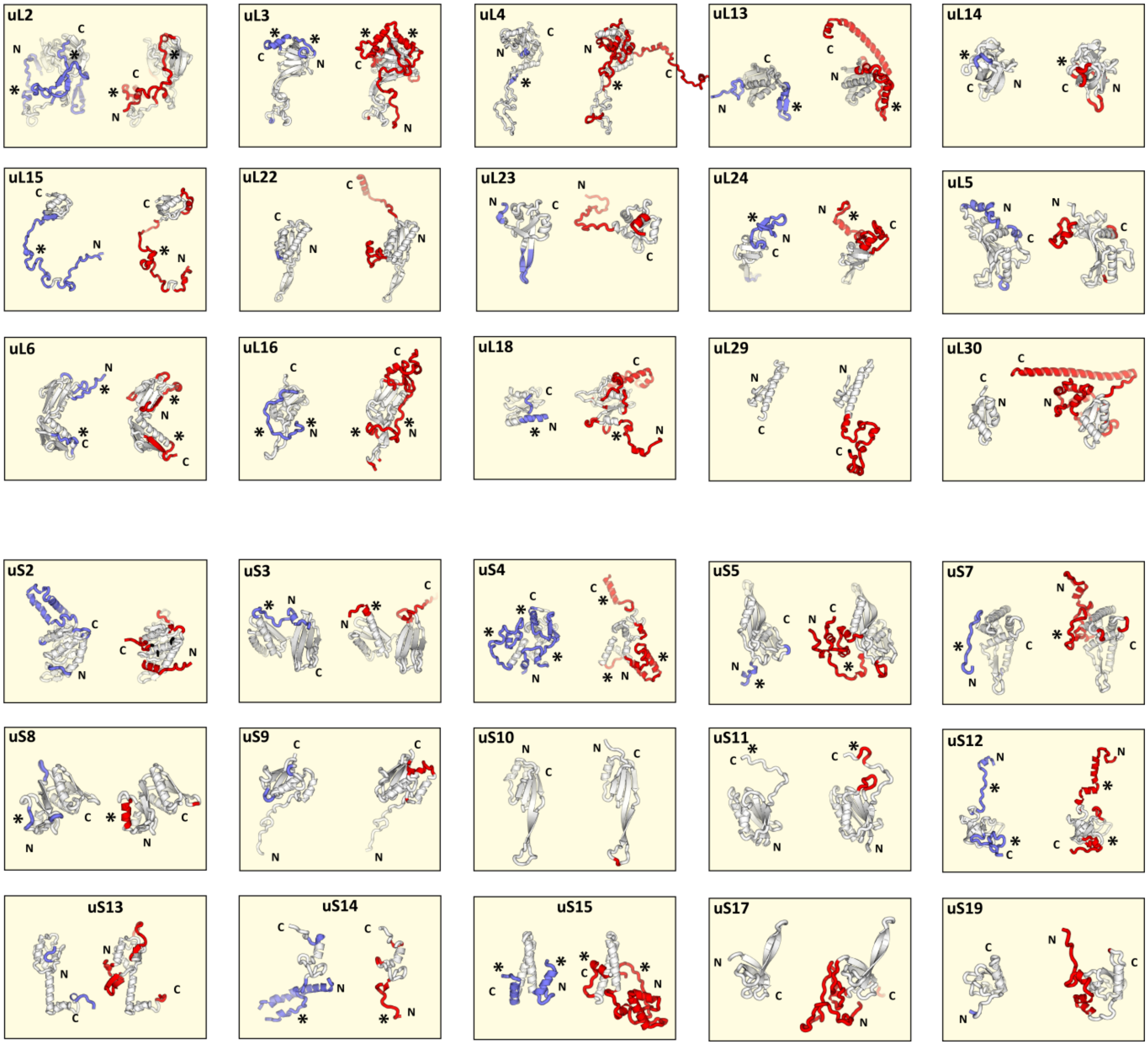

Supplementary Figure 2: Comprehensive comparison of proteins from bacterial and eukaryotic ribosomes reveals different folds of their seemingly conserved non-globular extensions. Panels show side-to-side comparison of homologous ribosomal proteins from the 70S *E. coli* (pdb-id 4WF1) and the 80S *S. cerevisiae* ribosomes (pdb-id 4V88). Each pair of structures was aligned by superposing conserved rRNA backbones within 6Å-distance around protein extensions. Asterisks point to the segments, which were previously considered as partially or fully conserved – according to sequence[10,11] or secondary-structure alignments[12]. Importantly, as shown in the (**Supplementary Figure 1**), these structural differences may potentially point to the location of the nuclear localization signals in eukaryotic ribosomal proteins.

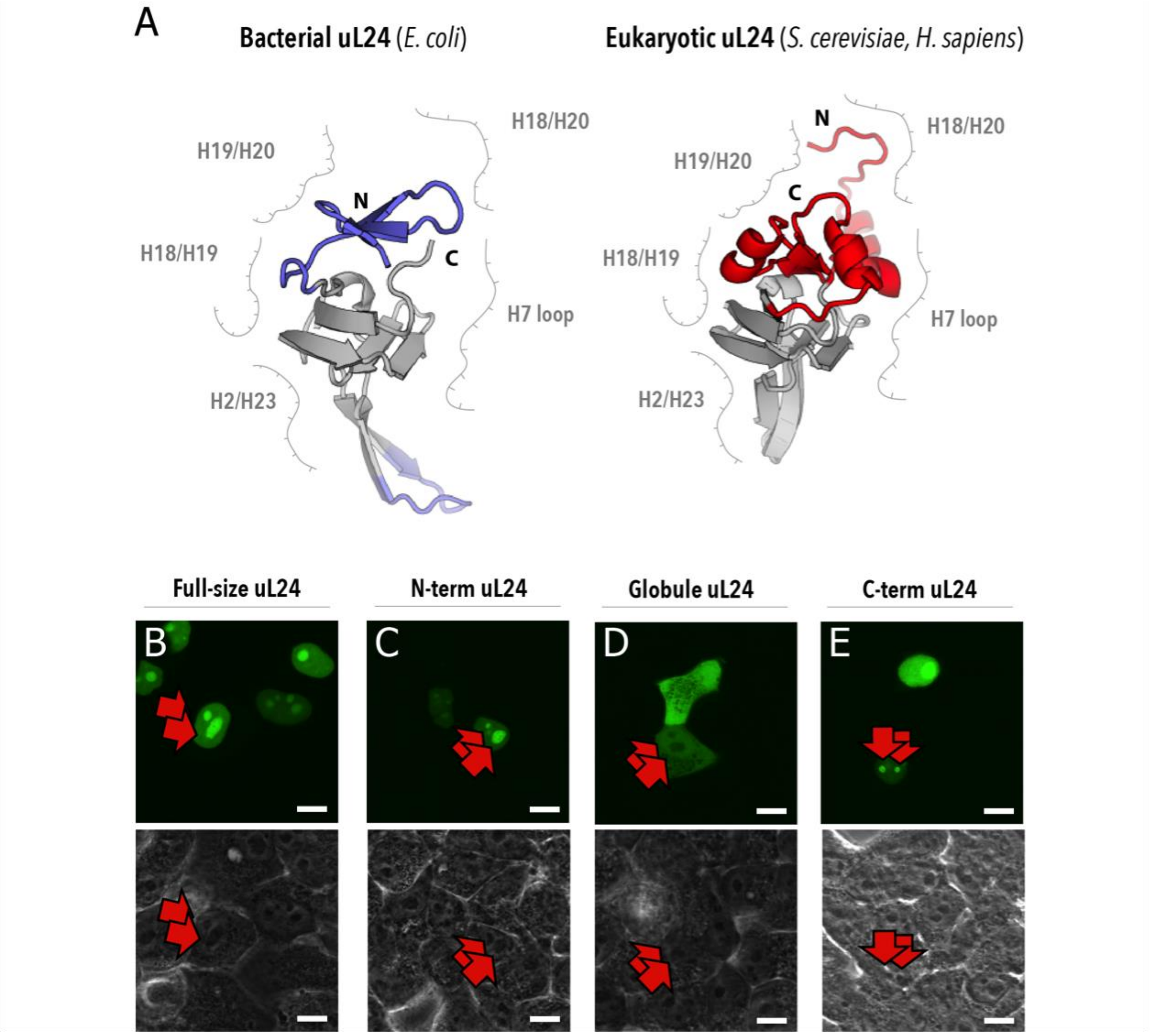

Supplementary Figure 3: Structure-guided identification of the NLSs in human ribosomal protein uL24. (**a**) Panels show structures of protein uL24 from *E. coli* and *S. cerevisiae* ribosomes, colored according to conservation, with conserved segments shown in grey and bacteria- and eukaryote-specific segments in blue and red, respectively. The N- and C-terminus of eukaryotic uL24 overlap with the N-terminus of bacterial uL24, but have different fold. The rRNA surrounding is shown schematically; labels correspond to the helices of 23S or 25S rRNA. (**b-e**) The panels show fluorescent (the top row) and phase-contrast (the bottom row) snapshots of human cell line HEK293, expressing eGFP-fusions with full-size or deletion mutants of human uL24. Red arrows point to nucleoli. All scale bars represent 10 μm. For each sample, eGFP localization was examined in 200 cells. Cells, in which eGFP distribution pattern was common for >95% of the analyzed population, were used for imaging. (**b**) uL24-eGFP; (**c**) N-terminal residues 1-49 uL24-eGFP; (**d**) the globular domain – residues 50-107 uL24-eGFP; (**e**) and the C-terminal 108-127 residues uL24-eGFP. The localization pattern indicates that the nuclear localization signals of human uL24 reside within its structurally remodeled N- and C-termini, which have different folds and size in bacterial uL24.

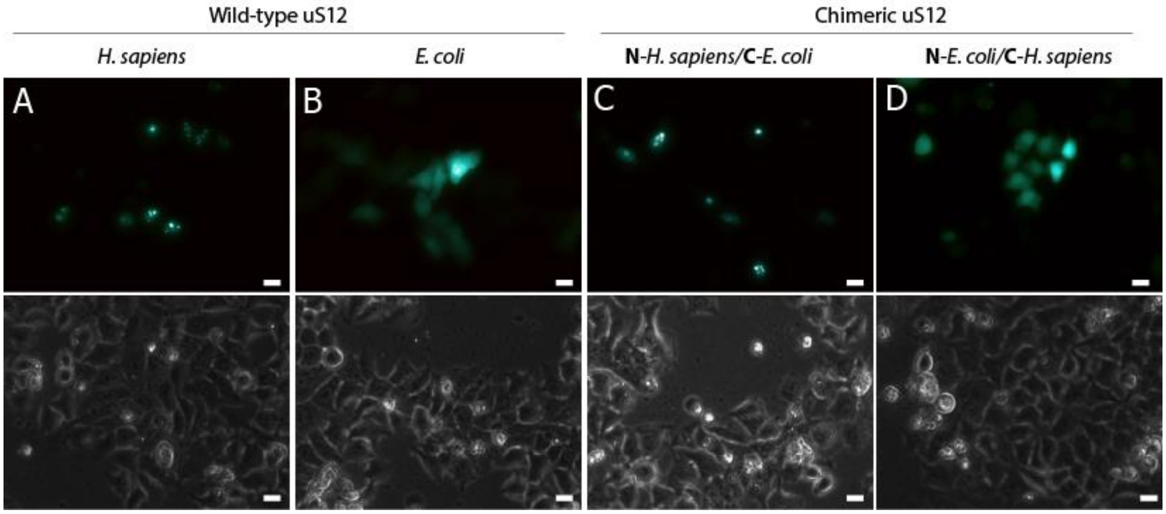

Supplementary Figure 4: NLS in human ribosomal protein uS12. The figure accompanies Figure 2 and shows a uniform pattern of intracellular eGFP distribution in each sample. (**a-d**) Panels show eGFP fluorescence (top panels) and phase-contrast (bottom panels) snapshots of human cell line HEK293, which express eGFP-fusions of human or *E. coli* protein uS12. All scale bars represent 20 μm. (**a**) Human uS12-eGFP fusion. (**b**) *E. coli* uS12-eGFP fusion. (**c**) A protein hybrid carrying the N-terminus of human uS12 and the globular domain of *E. coli* uS12. (**d**) A protein hybrid carrying the N-terminus of *E. coli* uS12 and the globular domain of human uS12.

A

Protein	Old name	Organism	Sequence	Refs	Tag used to monitor the nucleolar accumulation
**uS3**	S3	*S. cerevisiae*<br>*H. sapiens*	3-**KKRK**-7	1	eGFP at the C-terminus<br>(*S. cerevisiae*)
**uS4**	S9	*H. sapiens*	171-**GRVKRKNAKKGQ**-182	2	eGFP at the N- and C-termini
**uS8**	S22	*S. cerevisiae*	20-**GKRQVLIRP**-28	3	β-galactosidase at the N-terminus
**uL3**	L3	*S. cerevisiae*	2-**SHRKYEAPRHGHLGFLPRKRA**-21	4	β-galactosidase at the C-terminus
**uL13**	L13a	*H. sapiens*	59-**RRK**-61	5	HA-tag, position is not specified
**uL15**	L29	*S. cerevisiae*	6-**KTRKHRG**-13<br>23-**KHRKHPG**-29	6	β-galactosidase at the C-terminus
**uL18**	L5	*H. sapiens*<br>*X. laevis*	21-**RRRREGKTDYYARKRLV**-37<br>255-**KKPKKEVKKKR**-265	7	eGFP at the C-terminus
**uL23**	L23a, L25	*S .cerevisiae,*<br>*H. sapiens*	11-**KKAVVKG**-17<br>18-**TNGKKALKVRT**-28	8	β-galactosidase at the C-terminus<br>(*S. cerevisiae*)
**uL29**	L35	*H. sapiens*	71-**KGKKYQPKDLRAKKTRALRRALTKF**-95	9	GST at the C-terminus

B

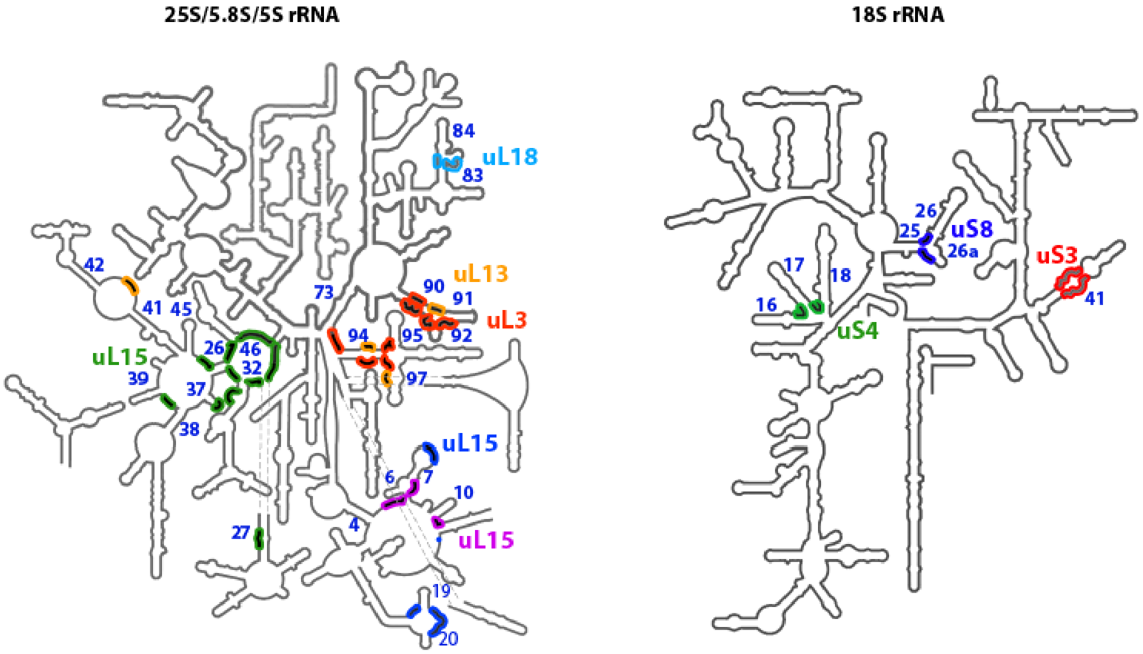

Supplementary Table 1: Previously identified NLSs in conserved ribosomal proteins, and their interactions with rRNA within the ribosome interior. (a) The table summarizes the primary structure of the known NLSs. Of note, proteins uL15, uL18 and uL23 carry two NLSs per protein. (**b**) The panel shows secondary-structure diagrams of 25S/5.8S/5S and 18S rRNA, which highlight rRNA contacts of the NLSs. Numbers correspond to the helices of rRNA. Within the ribosome interior, the NLSs are buried in rRNA and associate predominantly with single-stranded segments of rRNA, which are comprising internal loops and helical junctions, suggesting the role of NLSs in rRNA folding.

Supplementary references

1. Koch, B. et al. Yar1 protects the ribosomal protein Rps3 from aggregation. *J. Biol. Chem.* **287**, 21806-21815 (2012).
2. Lindstrom, M.S. Elucidation of motifs in ribosomal protein S9 that mediate its nucleolar localization and binding to NPM1/nucleophosmin. *PLoS One* **7**, e52476 (2012).
3. Timmers, A.C., Stuger, R., Schaap, P.J., van 't Riet, J. & Raue, H.A. Nuclear and nucleolar localization of Saccharomyces cerevisiae ribosomal proteins S22 and S25. *FEBS Lett.* **452**, 335-340 (1999).
4. Moreland, R.B., Nam, H.G., Hereford, L.M. & Fried, H.M. Identification of a nuclear localization signal of a yeast ribosomal protein. *Proc. Natl Acad. Sci. USA* **82**, 6561-6565 (1985).
5. Das, P. et al. Insights into the mechanism of ribosomal incorporation of mammalian L13a protein during ribosome biogenesis. *Mol. Cell. Biol.* **33**, 2829-2842 (2013).
6. Underwood, M.R. & Fried, H.M. Characterization of nuclear localizing sequences derived from yeast ribosomal protein L29. *EMBO J.* **9**, 91-99 (1990).
7. Rosorius, O. et al. Human ribosomal protein L5 contains defined nuclear localization and export signals. *J Biol Chem* **275**, 12061-12068 (2000).
8. Schaap, P.J., van't Riet, J., Woldringh, C.L. & Raue, H.A. Identification and functional analysis of the nuclear localization signals of ribosomal protein L25 from Saccharomyces cerevisiae. *J. Mol. Biol.* **221**, 225-237 (1991).
9. Chen, I.J., Wang, I.A., Tai, L.R. & Lin, A. The role of expansion segment of human ribosomal protein L35 in nuclear entry, translation activity, and endoplasmic reticulum docking. *Biochem. Cell. Biol.* **86**, 271-277 (2008).
10. Klinge, S., Voigts-Hoffmann, F., Leibundgut, M., Arpagaus, S. & Ban, N. Crystal structure of the eukaryotic 60S ribosomal subunit in complex with initiation factor 6. *Science* **334**, 941-948, (2011).
11. Rabl, J., Leibundgut, M., Ataide, S. F., Haag, A. & Ban, N. Crystal structure of the eukaryotic 40S ribosomal subunit in complex with initiation factor 1. *Science* **331**, 730-736, (2011).
12. Ben-Shem, A. *et al.* The structure of the eukaryotic ribosome at 3.0 A resolution. *Science* **334**, 1524-1529 (2011).
